# Efficient Performance of Electrostatic Spray-Deposited TiO_2_ Blocking Layers in Dye-Sensitized Solar Cells after Swift Heavy Ion Beam Irradiation

**DOI:** 10.1007/s11671-010-9763-2

**Published:** 2010-09-16

**Authors:** P Sudhagar, K Asokan, June Hyuk Jung, Yong-Gun Lee, Suil Park, Yong Soo Kang

**Affiliations:** 1 Center for Next Generation Dye-Sensitized Solar Cells, WCU Program, Department of Energy Engineering, Hanyang University, Seoul, 133-791, South Korea; 2 Inter-University Accelerator Centre, Aruna Asaf Ali Marg, New Delhi, 110 067, India; 3 School of Chemical and Biological Engineering, Seoul National University, Seoul, South Korea

**Keywords:** Titanium oxide, Interfaces, Impedance spectroscopy, Dye-sensitized solar cells, Ion beam irradiation

## Abstract

A compact TiO_2_ layer (~1.1 μm) prepared by electrostatic spray deposition (ESD) and swift heavy ion beam (SHI) irradiation using oxygen ions onto a fluorinated tin oxide (FTO) conducting substrate showed enhancement of photovoltaic performance in dye-sensitized solar cells (DSSCs). The short circuit current density (J_sc_ = 12.2 mA cm^-2^) of DSSCs was found to increase significantly when an ESD technique was applied for fabrication of the TiO_2_ blocking layer, compared to a conventional spin-coated layer (J_sc_ = 8.9 mA cm^-2^). When SHI irradiation of oxygen ions of fluence 1 × 10^13^ ions/cm^2^ was carried out on the ESD TiO_2_, it was found that the energy conversion efficiency improved mainly due to the increase in open circuit voltage of DSSCs. This increased energy conversion efficiency seems to be associated with improved electronic energy transfer by increasing the densification of the blocking layer and improving the adhesion between the blocking layer and the FTO substrate. The adhesion results from instantaneous local melting of the TiO_2_ particles. An increase in the electron transport from the blocking layer may also retard the electron recombination process due to the oxidized species present in the electrolyte. These findings from novel treatments using ESD and SHI irradiation techniques may provide a new tool to improve the photovoltaic performance of DSSCs.

## Introduction

Dye-sensitized solar cells (DSSCs) are a promising photovoltaic system for next generation solar cells that contain mesoporous nanocrystalline semiconductors like TiO_2_, ZnO and SnO_2_ as photoanodes anchored with dye molecules. These dye molecules serve as light harvesters [1-3]. It is believed that DSSCs are more cost effective than conventional solar cells due to their low production cost. Recently, intensive research activities have focused on enhancing the photoconversion efficiency of DSSCs by improving charge transport in the electronic interfaces such as (a) TiO_2_/transparent conducting oxide (b) TiO_2_/electrolyte (c) dye/TiO_2_ (d) dye/electrolyte and (e) electrolyte/counter electrode. For instance, electrons on either side of the TiO_2_ layer or in the transparent conducting oxide (TCO) such as fluorinated tin oxide (FTO) may recombine with the oxidized redox couples such as I_3_^-^. Electron recombination is one of the major factors that determine the high energy conversion efficiency (2e^-^+I_3_^-^ → 3I^-^) [4,5]. Therefore, there have been several different approaches to reduce or block the recombination of electrons on TCO and TiO_2_ layers to improve the energy conversion efficiency. Among the interfaces described previously, the one between TiO_2_/transparent conducting oxides faces severe recombination problems, since the porous nature of photoanodes results in uncovered sites on the TCO layer, resulting in sites for electron recombination with I_3_^-^ redox species in the electrolyte.

Considerable attention has been focused on the methods to reduce electron recombination at the interface between TCO substrate and electrolyte containing I_3_^-^. In order to overcome this recombination problem, a compact oxide layer (pore-free and dense) is commonly introduced between the mesoporous TiO_2_ and the TCO substrate, which blocks electron recombination with the electrolyte via a so-called blocking effect [6]. Furthermore, the blocking layer should provide good adhesive properties between the TCO and the mesoporous TiO_2_ layers to facilitate electron transport from the mesoporous TiO_2_ to the TCO layers. From this perspective, a variety of oxides have been investigated such as Nb_2_O_5 _[7], ZnO [8], MgO [9], Al_2_O_3 _[10] and SiO_2 _[11] in addition to TiO_2 _[12]. Different preparation techniques have been widely exploited to form blocking layers such as sol-gel [12], spin coating [13], sputtering [14,15] and spray-coating [16] techniques. Therefore, the formation of a blocking layer between mesoporous TiO_2_ and the TCO substrate has been investigated, which not only blocks electron recombination but also facilitates electron transport.

In this study, electrostatic spray deposition (ESD) was applied first for fabricating a TiO_2_ blocking layer, and swift heavy ion beam irradiation (SHI) was subsequently performed as a post-treatment, since ESD allows particle size and shape to be controlled by varying processing parameters such as the polymer concentration in the spray solution and applied voltage. Furthermore, a conventional electrospinning setup, in which the conducting FTO electrode directly connected to the electric circuit (negative terminal) may produce an electro-hydrodynamic field between a collector (FTO) and a sol injector (syringe), may improve adhesion between the sprayed particles and the FTO substrate. Particle growth achieved via ESD is more effective than that obtained by conventional spray pyrolysis [17] or spin coating. Chen et al. [18] reported nanostructured TiO_2_ films fabricated by ESD and studied their phase transformations by sintering. Zhang et al. [19] demonstrated the feasibility of ESD-derived uniform TiO_2_ particles in DSSCs and suggested that the electrical contact between the conducting substrate and TiO_2_ particle (electron transport layer) plays a crucial role in power conversion efficiency, since the presence and the removal of the polymer molecules in the ESD layer during sintering may result in poor contact among TiO_2_ nanoparticles and poor adhesion to conductive glass substrates. These will impose severe constraints on the electron transport from the mesoporous TiO_2_ layer to the FTO substrate. Therefore, an alternative post-treatment may be necessary to obtain a compact, thin blocking layer with good contact among TiO_2_ nanoparticles and good adhesion to the conductive glass substrates [20], resulting in rapid electron transport. SHI was employed as a post-treatment for improving both adhesion and contact. Recently, Singh et al. [21] reported that SHI irradiation improved the transmittance of conducting substrates (indium-doped tin oxide), and their performance was affected in DSSCs. The SHI method is based on the interactions of ions with solids, where the temperature around the trajectory of the ion increases remarkably. The shock waves, or so-called pressure waves, develop due to the temperature spike, which diffuses the heat radially in the target [22]. This thermal spike can generate local heat along TiO_2_ nanoparticles. When the temperature is greater than the melting temperature of TiO_2_ (~1,300^°^C), a liquid phase is formed in this specific region. This high temperature region cools down immediately due to very rapid heat transfer to the surroundings, resulting in solidification of the surface, specifically melted TiO_2_ nanoparticles [23] that form a highly adhesive TiO_2_ blocking layer with the FTO substrate. To best of our knowledge, this is the first report of its kind to apply the SHI irradiation technique for obtaining an efficient blocking layer in DSSCs. The performance of the SHI-irradiated blocking layer was investigated in comparison with the unirradiated (pristine) and conventional spin-coated TiO_2_ blocking layers.

## Experimental

The following procedure was used for the preparation of a TiO_2_ blocking layer on fluorinated tin oxide (FTO) substrates: 15 wt% poly(vinyl acetate) (PVAc) (Mn ~ 5,000,000) solution was prepared by dissolving PVAc in dimethyl formamide (DMF) and dropping it into a mixture containing 1 g of titanium isopropoxide and 0.5 g of acetic acid while stirring. The as-prepared TiO_2_ sol was electrosprayed onto a grounded FTO substrate at 17 kV with a constant distance of about 10 cm between FTO and the electrospray syringe at a flow rate of 1.0 ml/h. The resultant ESD TiO_2_ blocking layer was ~1.1 μm thick and was sintered at 450°C for 30 min in air. In order to prepare SHI-irradiated films, the as-prepared ESD TiO_2_ films were used without sintering.

SHI was conducted using 15 UD Pelletron tandem accelerator facilities available in the Materials Science Beamline at the Inter-University Accelerator Centre (IUAC), New Delhi, India. The vacuum of the experimental chamber was in the range of 10^-6^ torr. The TiO_2_ films, which act as blocking layers, were subjected to 100 MeV O ion irradiation with fluence of 1 × 10^13^ ions/cm^2^. The electronic and nuclear energy loss values for 100 MeV O ions in TiO_2_, calculated using the SRIM code simulation program (SRIM-2010) [24,25], were 1.284 × 10^2^ and 6.739 × 10^-2^ eV/Å, respectively. The range of O ions in this experiment is about 54.14 μm, indicating that the entire passage of ions in the film is dominated by electronic energy loss. Further experimental details were published elsewhere [26].

In order to compare the effect of the blocking layer, two kinds of DSSCs were assembled: (a) a *pristine cell* fabricated from the ESD TiO_2_ blocking layer and (b) a *SHI cell* using an irradiated ESD TiO_2_ blocking layer. In addition, *a reference cell* was fabricated from the TiO_2_ blocking layer prepared by conventional spin coating (Ti(IV) bis (ethyl acetonato)-diisopropoxide solution in 2 wt% of 1-butanol) and was also tested under identical experimental conditions. Further, TiO_2_ photoanodes thickness about ~6 μm were prepared on the TiO_2_ blocking layer using TiO_2_ paste (Solaronix) by a doctor blade technique [27] and subsequently sintered at 450°C for 30 min in air.

**Figure 1:**
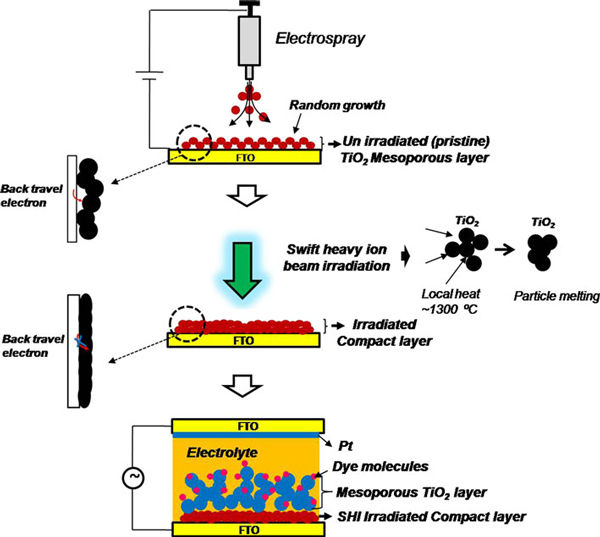
**Schematic of a electrostatic spray deposition of TiO_2_ compact layer, b SHI-irradiated TiO_2_ compact layer and, c SHI-irradiated TiO_2_ compact layer assisted DSSCs**.

N719 dye (di-tetrabutylammonium cis-bis(isothiocyanato)bis(2,2'-bipyridyl-4,4'-dicarboxylato)ruthenium(II)) was used to sensitize the TiO_2_ photo electrodes. The TiO_2_ electrodes were immersed overnight in a 0.3 mM dye solution containing a mixture of acetonitrile (ACN) and t-butyl alcohol (1:1 v/v) and dried at room temperature. A sandwich-type configuration was employed to measure the performance of the dye-sensitized solar cells, using a Pt-coated F-doped SnO_2_ film as a counter electrode and 0.5 M MPII (1-methyl-3-propylimidazolium iodide) with 0.05 M I_2_ in ACN as the electrolyte solution. Current–voltage characteristics of DSSCs were performed under 1 sun illumination (AM 1.5G, 100 mW cm^-2^) with a Newport (USA) solar simulator (300 W Xe source) and a Keithley 2,400 source meter (device area is 0.16 cm^2^). The different stages of the cell fabrication are schematically shown in Figure 1. Electrochemical impedance measurements were carried out using a potentiostat (IM6 ZAHNER) equipped with a frequency response analyzer (Thales) in the frequency range of 0.1 Hz–1,000 kHz. The results were analyzed with an equivalent circuit model for interpreting the characteristics of the DSSCs. Incident photon-to-current conversion efficiency (IPCE) of DSSCs was measured using PV Measurements Inc. (Model QEX7) with bias illumination with reference to the calibrated silicon diode.

The surface morphologies of the TiO_2_ thin films before and after SHI irradiation were studied by field-emission scanning electron microscopy (JEOL-JSM 6330F). The crystalline phases of the TiO_2_ films were determined by X-ray diffraction (XRD) using a diffractometer (Rigagu Denki Japan) with CuKα radiation. The conductivity of the samples was studied via the two-probe method.

## Results and Discussion

**Figure 2:**
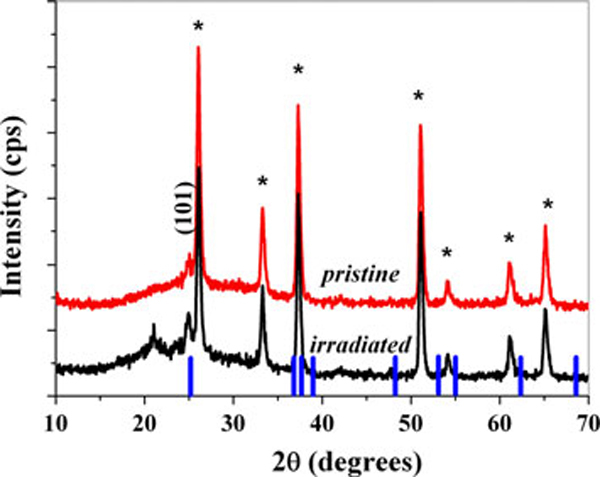
**X-ray diffraction spectra**. (Note that * indicated in the XRD spectra is indicated the crystalline contribution from FTO substrate.) Standard peak position (JCPDS 21-1272) of the TiO_2_ anatase phase is given in *vertical lines*.

Figure 2 shows the X-ray diffraction spectra of the ESD pristine and the SHI-irradiated TiO_2_ layers. Hereafter, the SHI-irradiated TiO_2_ layer is referred to as a layer formed by the ESD first and subsequently SHI-irradiated techniques. The characteristic peak observed at ~25.3° in both the films indicated the presence of an anatase phase of TiO_2_ (JCPDS 21-1272). The increase in the relative peak intensities observed in the SHI-irradiated sample shows that the SHI irradiation induced crystallization when compared to the as-prepared pristine ESD TiO_2_ films. The average grain size of the SHI-irradiated TiO_2_ films was found to be about 47 nm as estimated from Scherrer's equation. The significant additional peak exhibited in the SHI-irradiated sample is not clearly understood.

**Figure 3:**
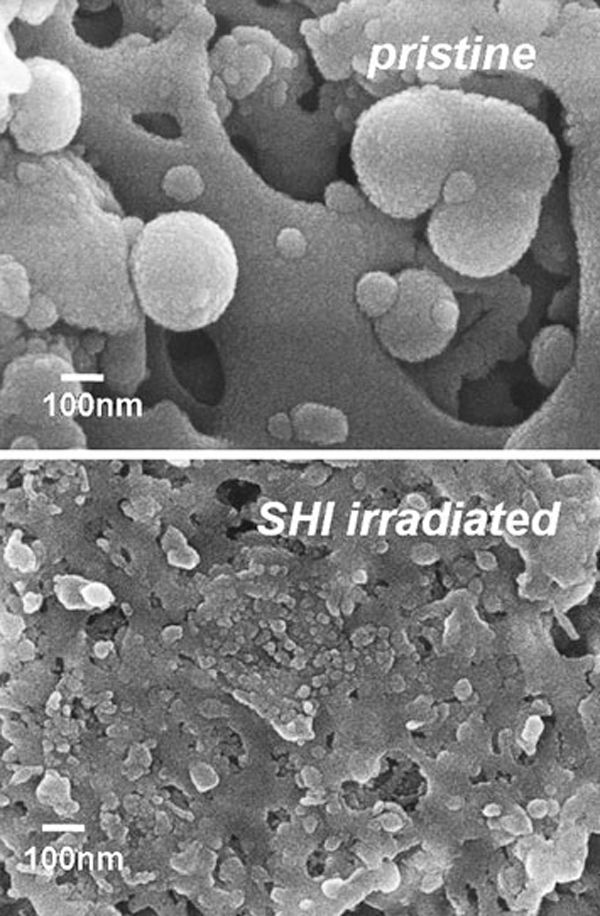
**Scanning electron microscopy images of pristine and O_2_ ion-irradiated TiO_2_ compact layer**.

**Figure 4:**
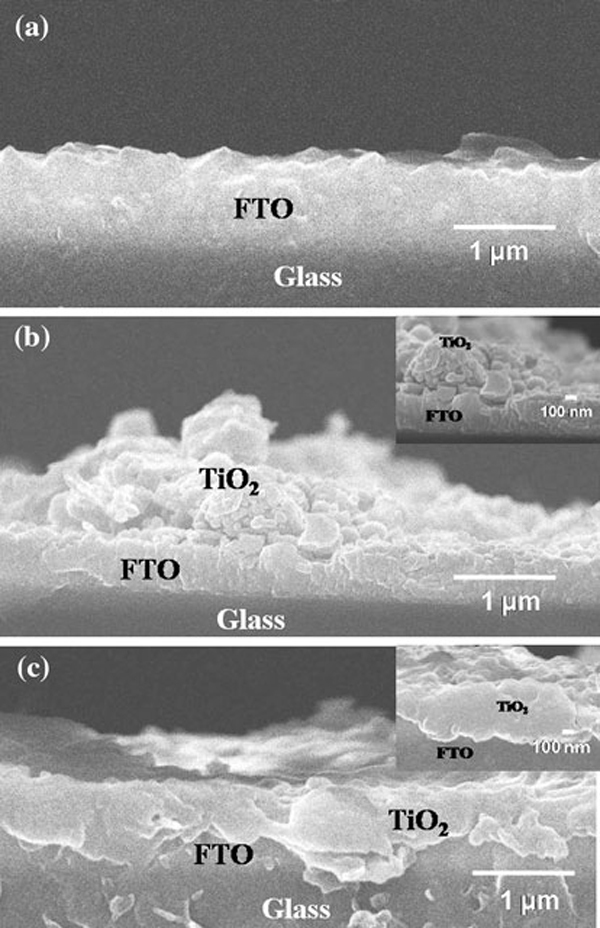
**Cross-sectional FE-SEM images of a bare FTO substrate, b pristine TiO_2_/FTO, and c O_2_ ion-irradiated TiO_2_/FTO**. The thickness of the pristine and irradiated TiO_2_ was about 1.1 and 0.67 μm, respectively. (Inset: images in 100 nm scale.)

Surface morphologies of the pristine and the SHI-irradiated TiO_2_ films are presented in Figure 3. The electrosprayed TiO_2_ films reveal an aggregation pattern, and the spherical particles form an interconnected porous framework of nano-sized building blocks (Figure 3). The observed nano-aggregated particles may be ascribed to the existence of a Coulumbic force lower than the stretching force resulting from weak repulsion between adjacent spray droplets. Under SHI irradiation, these nano-aggregated TiO_2_ particles melted and solidified on the FTO substrate and consequently formed a rather flat, nonporous structure with the FTO layer (see Figure 3). This results in a compact interface at FTO/TiO_2_ for both blocking electron recombination and increasing electronic transport. The fragmentation of the aggregated particles into smaller grains under SHI irradiation can be explained by a thermal spike model. If a large amount of energy is deposited by the projectile ions to the electronic subsystem of the target material, this energy can be shared among electrons by electron–electron coupling and later transferred quickly to the surrounding lattice through electron–phonon coupling. Thus, a sudden temperature rise on the time scale of 10^-12^ s along the ion track resulted in a molten state. The subsequent heat transfer to the surrounding lattice results in resolidification of this molten liquid phase. If this cooling rate slows to a critical value, nucleation of crystalline phases can be expected along the ion trajectory [28,29]. Therefore, we speculate that the surface of the TiO_2_ particles may undergo an ion-beam-induced molten state in a short duration of time (10^-12^ s). These molten state particles were attached with FTO substrate, enhancing the inter-particle connectivity (compact) to improve the conductivity of the film. The measured conductivity of the pristine and the SHI-irradiated TiO_2_ films found to be 2.31 × 10^-2^ and 1.2 Scm^-1^, respectively, indicating large improvement in the electron conductivity. Cross-sectional SEM images of the pristine and the SHI-irradiated TiO_2_ films are illustrated in Figure 4. Figure 4b suggests that the pristine ESD TiO_2_ layer has nano-aggregates and an inhomogeneous interface (contact) with the FTO layer, mostly due to the removal of polymer templates from ESD coating during sintering treatment. The observed inhomogeneous TiO_2_/FTO interface in the pristine sample was further compressed by SHI irradiation using O_2_ ions. This interface modification was confirmed by Figure 4c, showing that the TiO_2_ particles adhered well to the FTO layer. The thickness of the pristine film, ~1.1 μm, was reduced to ~0.67 μm after O ion irradiation. This is ascribed to the compact nature of TiO_2_ film formed by SHI irradiation. It is noteworthy to mention that improving the compact nature of the TiO_2_ blocking layer upon SHI irradiation can facilitate electron transport and also reduce electron recombination back to the electrolyte.

**Figure 5:**
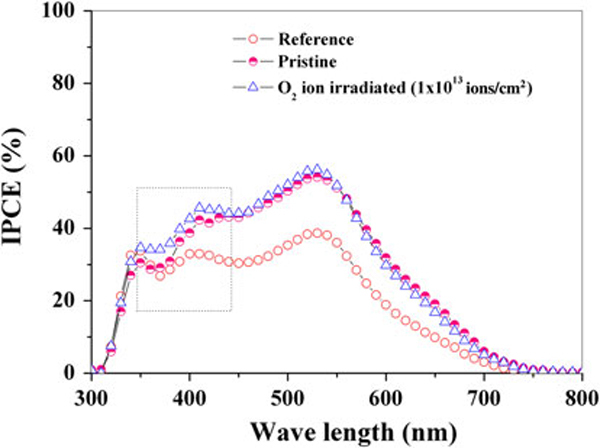
**IPCE spectra of DSSCs using different TiO_2_*blocking layers***.

As shown in Figure 5, the ESD TiO_2_ blocking layer DSSC (*pristine cell*) shows higher IPCE (maximum up to about ~53% at 530–540 nm) than the *reference cell* over the whole range of light wavelengths. This clearly demonstrates a ~16% improvement in external quantum efficiency from reducing the electron losses at FTO/TiO_2_ interfaces. It appears that the ESD is more efficient than the spin coating in terms of improving IPCE due to the formation of continuous films. Further, substantial improvement in IPCE was identified at lower wavelengths (380–420 nm), attributable to the SHI irradiation on the TiO_2_ blocking layer. The IPCE can be rationalized using the following relation [30],

(3) $$\text{IPCE}\left(\lambda \right)=A\varphi_{inj}\eta_{coll}$$

where *A* is the absorptivity indicating the fraction of incident light absorbed by the dye molecules, *φ*_*inj*_ is the injection efficiency of dye molecules into the TiO_2_ conduction band, and *η*_*coll*_ is the collection efficiency. The parameters A and *φ*_*inj*_ are directly related to dye loading on the TiO_2_ surface. In the present work, we have controlled similar dye loading in the *reference*, the *pristine* and the *SHI-irradiated electrodes*, as verified with a dye removal test using 1 M aqueous NaOH solution. Therefore, *A* and *φ*_*inj*_, of all these samples can be treated to be equal, and the change in the IPCE is related to the improvement in *η*_*coll*_. This improvement in *η*_*coll*_ under SHI irradiation can be ascribed to (a) better adhesion of the TiO_2_ blocking layer with the TCO substrate and (b) enhanced contact among TiO_2_ particles. Hence, it is expected that the SHI-irradiated blocking layer may result in higher photoconversion efficiency.

**Figure 6:**
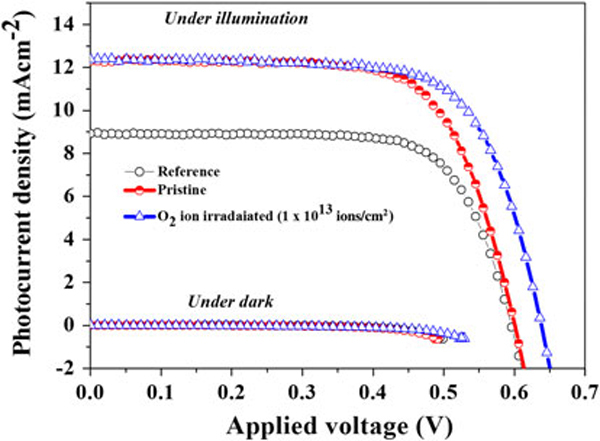
**J-V measurements under a light illumination (100 mW cm^-2^) along with b dark condition (lower part of the spectrum)**.

Figure 6 shows the photocurrent density–voltage (J-V) characteristics measured under 1 sun (100 mW cm^-2^ AM 1.5) and dark conditions. The photovoltaic parameters were estimated from Figure 6 and are summarized in Table 1. The photocurrent density (J_sc_) was increased from 8.9 to 12.2 mA cm^-2^, and the overall efficiency (η) was markedly improved from 3.8 to 5.1% by replacing the ESD TiO_2_ compact layer, compared to the conventionally spin-coated blocking layer. This might be attributed to the highly compact nature of the ESD films, which provide more effective pathways for electrons. As a result, electrons can be collected faster at the TCO and transferred to the external circuit, resulting in improvement in the photovoltaic performance. However, there is no appreciable change in the open circuit voltage (V_oc_) between these samples. When the ESD cell was treated with SHI irradiation, the open circuit voltage was further improved from 0.60 to 0.63 V, and consequently, the overall energy conversion efficiency improved from 5.1 to 5.5%. This may be because of the SHI irradiation, which melted TiO_2_ particles and thereby improved electrical contact with the FTO substrate (denser and more compact) and among TiO_2_ particles. This clearly demonstrates that the SHI irradiation enhances the blocking effect of electron recombination and creates a facilitating effect on electron transport.

**Table 1:** Influence of TiO_2_ blocking layer on photovoltaic parameters of DSSCs

Sample	V_oc_ (V)	J_sc_ (mA cm^-2^)	F.F (%)	Efficiency (%)
Reference	0.59	8.9	71.9	3.8
Pristine	0.60	12.2	69.3	5.1
O_2_ ion irradiated (1 × 10^13^ ions/cm^2^)	0.63	12.3	69.9	5.5

A comparison of dark currents between the investigated cells provides qualitative information about the electron recombination process [31]. In DSSCs, preventing the recapture of photoinjected electrons by I_3_^-^ is vital to obtain a high open circuit photovoltage. By inserting the blocking layer between the FTO substrate and the TiO_2_ mesoporous layer, the reaction possibilities of I_3_^-^ with the photoinjected electrons on the FTO substrate are significantly hindered, as demonstrated by the reduced dark current [31]. Here, the dark current–voltage curves of the DSSCs using different blocking layers are presented in the lower part of Figure 6. The less dark current observed in the *SHI-irradiated cell* compared with the *pristine cell* may be attributed to the better electrical contact between the blocking layer and the FTO substrate, and the compact nature of the blocking layer as well. Furthermore, during SHI irradiation, it is expected that Sn^4+^ particles from the FTO layer may fuse with the TiO_2_ layer occupying the oxygen vacancies in TiO_2_, thus lowering the Fermi level of TiO_2_. For instance, the Fermi level position of the Sn-doped TiO_2_ layer is lower than that of the TiO_2_ mesoporous layer, which is favorable for fast electron injection from mesoporous TiO_2_ particles to the conducting substrate [32].

**Figure 7:**
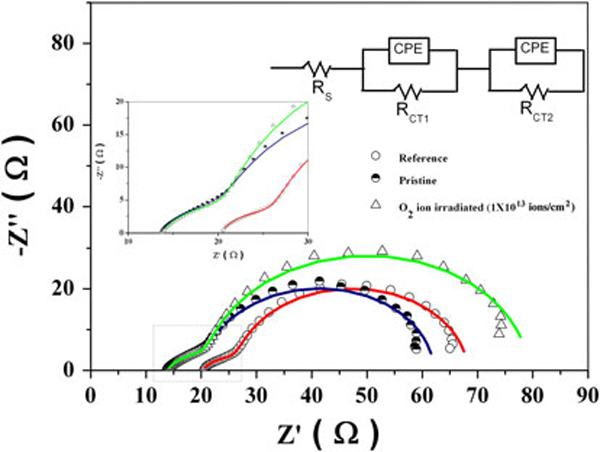
**Nyquist spectra (measured under light illumination (100 mW cm^-2^)) of DSSCs**. The inset represents the impedance spectra expanded in the high frequency ranges. The scattered points are experimental data, and the *solid lines* are the *fitting curves*.

Electrochemical impedance spectroscopy (EIS) provides valuable information on the kinetics of electron transport in the DSSCs with deeper understanding of the interfacial reactions at FTO/TiO_2 _[33] and therefore was employed to decipher the blocking layer effect in DSSCs. Figure 7 shows the Nyquist plots of the electrochemical impedance spectra. Their equivalent circuit is given as an inset in the figure. The charge transfer resistances R_CT1_ and R_CT2_ represent the resistances at the Pt/FTO and TiO_2_/dye/electrolyte interfaces, respectively. The electrochemical parameters were estimated by fitting experimental data with the equivalent circuit (inset of Figure 7) [34] and are summarized in Table 2.

**Table 2:** Influence of TiO_2_ blocking layer on electrochemical parameters of DSSCs

Sample	Rs (Ω)	R_CT1_ (Ω)	R_CT2_ (Ω)
Reference	20.3	6.7	41.9
Pristine	13.6	8.6	39.8
O_2_ ion-irradiated (1 × 10^13^ ions/cm^2^)	13.9	7.9	57.3

The series resistance, R_s_, was decreased markedly in the case of the pristine and O ion-irradiated electrodes, compared to the reference electrode. This is mostly associated with better electron transfer through the blocking layer due to better contact and better adhesion. The R_CT2_ value for *SHI cells* was increased markedly compared to the reference and the pristine electrodes. The increased R_CT2_ value may be mostly due to the fast electron transfer through the blocking layer. Hence, the increased electron transfer leads to lowering electron concentration of TiO_2_ mesoporous particles, which is responsible for observed high R_CT2_ (57.3 Ω) values in the O ion-irradiated sample.

The results described above suggest that contact among nanoparticles and the adhesion properties of a blocking layer with an FTO substrate may improve the performance of dye-sensitized solar cells. Further studies using different ion energies and fluence may further explain the role of electronic energy loss on these devices and allow development of precise control of the blocking layer.

## Conclusions

An electrostatic spray deposition (ESD) technique followed by SHI irradiation using 100 MeV oxygen ions resulted in the formation of an efficient, dense TiO_2_ blocking layer between the TiO_2_ particle layer and the TCO substrate. The blocking layer promotes charge transport from the TiO_2_ layer to the TCO substrate by modifying the TCO/TiO_2_ interfaces and causes effective electrical contact between the two layers. The formation of an effective, compact blocking layer was possible due to instantaneous surface melting of the ESD TiO_2_ nanoparticles associated with a local temperature rise upon oxygen ion irradiation. Energy conversion efficiency was improved to a large extent (η = 5.5%), compared to that of the conventional blocking layer (η = 3.8%), mainly due to the increase in electron transport through the blocking layer, resulting from better contact among TiO_2_ nanoparticles and better adhesion with the TCO substrate.

## References

[B1] Gratzel M Nature 2001 414 338 10.1038/35104607 11713540 11713540

[B2] Quintana M Edvinsson T Hagfeldt A Boschloo G J Phys Chem C 2007 111 1035 10.1021/jp065948f

[B3] Fukai Y Kondo Y Mori S Suzukia E Electrochem Comm 2007 9 1439 10.1016/j.elecom.2007.01.054

[B4] Cameron PJ Peter LM J Phys Chem B 2003 107 14394 10.1021/jp030790+

[B5] Durrant JR Haque SA Palomares E Coord Chem Rev 2004 248 1247 10.1016/j.ccr.2004.03.014

[B6] Cameron PJ Peter LM J Phys Chem 2005 109 7392 10.1021/jp0407270 16851846

[B7] Xia J Masaki N Jiang K Yanagida S J Phys Chem C 2007 111 8092 10.1021/jp0707384

[B8] Zhang Y Wu L Li Y Xie E J Phys D Appl Phys 2009 42 085105 10.1088/0022-3727/42/8/085105

[B9] Jung HS Lee J-K Nastasi M Lee S-W Kim JY Park JS Hong KS Langmuir 2005 21 10332 10.1021/la051807d 16262288 16262288

[B10] Law M Greene LE Radenovic A Kuykendall T Liphardt J Yang P J Phys Chem B 2006 110 22652 10.1021/jp0648644 17092013 17092013

[B11] Nguyen V Lee H-C Khan MA Yang O-B Sol Energy 2007 81 529 10.1016/j.solener.2006.07.008

[B12] Hart JN Menzies D Cheng Y-B Simon GP Spiccia L Chimie CR 2006 9 622

[B13] Papageorgiou N Maier WF Grätzel M J Electrochem Soc 1997 144 876 10.1149/1.1837502

[B14] Hossain MF Biswas S Takahashi T Thin Solid Films 2008 517 1294 10.1016/j.tsf.2008.06.027

[B15] Waita SM Aduda BO Mwabora JM Niklasson GA Granqvist CG Boschloo G J Electroanal Chem 2009 637 79 10.1016/j.jelechem.2009.10.004

[B16] Peng B Jungmann G Jager C Haarer D Schmidt H-W Thelakkat M Coord Chem Rev 2004 248 1479 10.1016/j.ccr.2004.02.008

[B17] Tachibana Y Umekita K Otsuka Y Kuwabata S J Phys D Appl Phys 2008 41 102002 10.1088/0022-3727/41/10/102002

[B18] Zhang Y Wu L Xie E Duan H Han W Zhao J J Power Sour 2009 189 1256 10.1016/j.jpowsour.2009.01.023

[B19] Chen CH Kelder EM Schoonman J Thin Solid Films 1999 342 35 10.1016/S0040-6090(98)01160-2

[B20] Fujihara K Kumar A Jose R Ramakrishna S Uchida S Nanotechnology 2007 18 365709 10.1088/0957-4484/18/36/365709

[B21] Singh HK Agarwal DC Chavhan PM Mehrad RM Aggarwal S Kulriya PK Tripathi A Avasthi DK Nucl Instrum Methods Phys Res Sect B Beam Interact Materials Atoms10.1016/j.physletb.2003.10.071

[B22] Kumar V Kumar R Locha SP Singh N Nucl Instr Meth B 2007 262 194 10.1016/j.nimb.2007.06.006

[B23] Thakurdesai M Kanjilal D Bhattacharyya V Semicond Sci Technol 2009 24 085023 10.1088/0268-1242/24/8/085023

[B24] Zeigler JF Biersack JP Littmark U The Stopping and Range of Ions in Solids 1985 1 Pergamon, New York

[B25] http://www.srim.org/

[B26] Chandramohan S Sathyamoorthy R Sudhagar P Kanjilal D Kabiraj D Asokan K Ganesan V Shripathi T Deshpande UP Appl Phys A 2009 94 703 10.1007/s00339-008-4866-7

[B27] Chena W Suna X Caia Q Weng D Lia H Electrochem Commun 2007 9 382 10.1016/j.elecom.2006.10.002

[B28] Trinkaus H Ryazanov AI Phys Rev Lett 1995 74 5072 10.1103/PhysRevLett.74.5072 10058676 10058676

[B29] Szenes G Phys Rev B 1995 51 8026 10.1103/PhysRevB.51.8026 9977411

[B30] Nazeeruddin MK Kay A Rodicio I Humphry-Baker R Mueller E Liska P Vlachopoulos N Graetzel M J Am Chem Soc 1993 115 6382 10.1021/ja00067a063

[B31] Ito S Liska P Comte P Charvet R Pechy P Bach U Schmidt-Mende L Zakeeruddin SM Kay A Nazeeruddin MK Grätzel M Chem Commun 2005 4351 10.1039/b505718c 16113745

[B32] Cao Y He T Chen Y Cao Y J Phys Chem C 2010 114 3627 10.1021/jp100786x

[B33] Fabregat-Santiago AF Bisquert J Palomares E Otero L Kuang D Zakeeruddin SM Grätzel M J Phys Chem C 2007 111 6550 10.1021/jp066178a

[B34] Wang Q Moser JE Grätzel M J Phys Chem B 2005 109 14945 10.1021/jp052768h 16852893 16852893

